# Applying cognitive load theory to medical education: construct and measurement challenges

**DOI:** 10.1007/s40037-015-0193-9

**Published:** 2015-05-28

**Authors:** John Q. Young, Justin L. Sewell

**Affiliations:** 1 Department of Psychiatry, Hofstra North Shore-LIJ School of Medicine, 75–59 263rd Street, Kaufman 217A, Glen Oaks, NY 11004 US; 2 Department of Medicine, UCSF School of Medicine, San Francisco, US

Leppink and Van den Heuvel’s [1] article explores cognitive load theory (CLT), a framework for learning that has recently received increased attention in medical education [2]. CLT builds upon a model of human memory developed by Atkinson and Shiffrin [3] that includes three primary sub-systems (sensory, working, and long-term memory). Unlike sensory and long-term memory, working memory is severely constrained—it can only hold a limited number of information elements at any given moment [4]. Because of this constraint, CLT identifies working memory as a ‘bottleneck’ for learning.

With working memory as the rate limiting step, CLT focuses on the cognitive load that learning tasks impose on the working memory. Originally, CLT described two types of cognitive load: intrinsic load (essential to the learning task) and extraneous load (non-essential to the learning task and often induced by poor instructional design) [5]. Later, CLT researchers proposed a third type, germane load, imposed by the deliberate use of cognitive strategies to enhance schema formation and automation. Overall, instructional techniques developed by CLT aim to optimize learning by minimizing extraneous load, matching intrinsic load to the developmental stage of the learner, and promoting germane load [6]. Leppink and Van den Heuvel’s article describes several excellent strategies to optimize intrinsic load and minimize extraneous load.

As noted by the authors, there is legitimate disagreement about the construct of germane cognitive load and how it relates to intrinsic load. However, we do not agree with Leppink and Van den Heuvel’s rejection of germane load as an independent construct. Four of six prior studies performed by Leppink [1, 7] produced three-factor solutions in factor analysis for cognitive load sub-types. Factor analysis from our own research (manuscripts in preparation) has also clearly shown a third factor when looking at cognitive load during two different clinical procedures, colonoscopy and patient handovers. Moreover, the inconsistent correlation of germane load with learning found in the studies to date may in part be due to assessing the learning outcome too soon after the intervention. The effect of germane load on learning may not be captured within hours of a learning task (the time frame used in Leppink’s prior studies). Thus, a third factor seems to exist. Reconceiving this third factor as ‘subjective judgment of learning’ seems premature at this point. Future studies utilizing CLT should focus on further specifying, characterizing, and refining the third factor and, in so doing, assess its correlation with learning at a later time horizon.

We believe that there is a reasonable theoretical argument for the construct of germane load. Some understand intrinsic load as related to task performance and germane load as related to task learning. In this view, germane load encompasses the mental activities related to schema construction and automation. Others argue that the construct of intrinsic load should include schema acquisition and that germane load should be limited to additional activities that enhance learning such as the conscious application of learning strategies (e.g., compare and contrast) [8]. Still others prefer to conceive of intrinsic load as including all of the activities attributed by others to germane load. This viewpoint rejects germane load as an independent construct. Because few researchers have sought to separately measure germane load, there is a relative paucity of data to specifically support any of these three definitions.

We envision intrinsic load as a relatively static characteristic of a learning task—its inherent difficulty is not under the control of the learner and can only be modified by simplifying the task or increasing the knowledge of the learner. Alternatively, we envision germane load—that is, effort to construct and refine learning schema—as being largely under the control of the learner. As an example, two learners with similar prior experience, undergoing the same learning task in the same format (thus equalizing intrinsic and extraneous load between the two learners), could experience significantly different levels of germane load depending on motivation, effort, and the possession of metacognitive skills. Until further research provides additional information about germane load, we maintain that there remains value in differentiating between the cognitive load associated with performing the task (i.e., manipulating in working memory the relevant information elements) and load associated with additional, conscious, and therefore modifiable, effort aimed at refining schema (e.g., monitoring one’s own understanding, etc.). In fact, we note the potential overlap between germane load as ‘conscious and effortful use of learning strategies’ and the constructs of motivation (‘effortful’) and meta-cognition [9]. Thus, germane load is likely inadequately specified by our current models.

In addition to the forgoing ‘construct’ challenges, there are also ‘measurement’ challenges associated with studies of CLT. The use of instruments that measure only overall load has limited testing and application of the theory. For example, integrating visual and written information has been shown to reduce overall load and improve learning [10]. Some have assumed that this occurs due to decreased extraneous load [11] while others have argued that the benefit of data integration is also mediated by increased germane load [12]. The absence of measures of specific load types permits competing and sometimes contradictory explanations to exist in parallel. The development of instruments that can measure the different cognitive load sub-types will not only help with addressing the ‘construct’ challenges above, but are essential to identifying how instructional techniques differentially impact the sub-types. Future instruments to measure cognitive load should explore the inclusion of metacognition concepts given the similarities between the concept of germane load and metacognition (e.g., monitoring understanding and adapting action in real time). To date, there is no published literature on measuring cognitive load sub-types during medical tasks; our own aforementioned work should represent an initial foray into this area.

We strongly agree with Leppink and Van den Heuvel’s suggestion that future research should address the role of emotion in CLT. For example, we know that positive emotions are associated with deeper processing, but there are many unknowns. How does the emotional state of the learner affect available working memory resources? And what kind of strategies can most effectively modulate this? To date, CLT has understood extraneous load as related to sources external to the learner such as background noise or how data are presented on a slide. We note that a learner’s anxiety, self-consciousness at being observed by an attending, fatigue, internal thoughts and distractions, or other factors internal to the learner may contribute to extraneous load and therefore ‘consume’ working memory resources. And the impact of emotion may go beyond extraneous load. Future research will hopefully lead to a specification of load sub-types that incorporates the impact of internal and affective processes.

The specific instructional techniques described by Leppink and Van den Heuvel are excellent. In CLT, extraneous load strategies have focused on reducing or eliminating the source. Some sources of extraneous load may be unavoidable (e.g., certain types of interruptions or internally generated distraction). We wonder if there are ‘teachable’ skills—perhaps, mindfulness or concentration maintenance skills—that could in effect reduce the WM impact of a given distraction. This is a potential area for future research. Of note, the techniques of ‘use worked examples’, ‘use completion tasks’, and ‘start with non-specific goals’, are traditionally understood as means to reduce extraneous load by reducing ‘problem solving search’. The classification used by Leppink and Van den Heuvel is consistent with this approach. Yet, these techniques could alternatively be understood as different examples of ‘part-task practice’—a strategy for reducing intrinsic load. This highlights the point that the distinction between extraneous and intrinsic load is contextual and highly dependent on how one defines the goal of the learning task. One task’s extraneous load is another’s intrinsic load. We also agree with Leppink and Van den Heuvel’s recommendation to decrease instructional support as the learner cycles from low to high task complexity. We do note that the role of task fidelity may vary, depending on the type of task. For example, in tasks such as the diagnostic assessment of a patient in a primary care setting, the patient can be protected via direct supervision which allows for high fidelity tasks for learners very early in training. Alternatively, in the case of surgical procedures, high-fidelity tasks for early learners could pose patient safety risks, and lower fidelity tasks (i.e., retraction, part-task trainers) are likely more appropriate for early surgical learners. These and many other examples highlight the variable role of task fidelity depending on the learning setting.

Despite these challenges, CLT is a well-developed framework that has the potential to make significant contributions to medical education. When a learner’s working memory is overloaded, performance is impaired, errors occur, and patient harm may ensue. In designing medical learning, attending to working memory and the strategies of managing cognitive load are highly relevant. As we develop more accurate measures of cognitive load sub-types, we will be better able to leverage CLT’s understanding of working memory to optimize learning for our learners and outcomes for patients.

## Declaration of Interest

None.

## Notes to Contributors

Not required for Commentary.

## Grant support

None.
